# Functional and Pathogenic Roles of Retroviral Antisense Transcripts

**DOI:** 10.3389/fimmu.2022.875211

**Published:** 2022-04-29

**Authors:** Kosuke Toyoda, Masao Matsuoka

**Affiliations:** Department of Hematology, Rheumatology and Infectious Diseases, Faculty of Life Sciences, Kumamoto University, Kumamoto, Japan

**Keywords:** human T-cell leukemia virus type 1 (HTLV-1), HTLV-1 bZIP factor (HBZ), human immunodeficiency virus type 1 (HIV-1), bovine leukemia virus (BLV), long non-coding RNA (lncRNA)

## Abstract

Exogenous retroviruses such as human immunodeficiency virus type 1 (HIV-1), human T-cell leukemia virus type 1 (HTLV-1) and bovine leukemia virus (BLV) can cause various diseases including immunodeficiency, inflammatory diseases and hematologic malignancies. These retroviruses persistently infect their hosts. Therefore, they need to evade host immune surveillance. One way in which these viruses might avoid immune detection is to utilize functional RNAs, rather than proteins, for certain activities, because RNAs are not recognized by the host immune system. HTLV-1 encodes the *HTLV-1 bZIP factor* (*HBZ*) gene in the antisense strand of the provirus. The HBZ protein is constantly expressed in HTLV-1 carriers and patients with adult T-cell leukemia-lymphoma, and it plays critical roles in pathogenesis. However, *HBZ* not only encodes this protein, but also functions as mRNA. Thus, *HBZ* gene mRNA is bifunctional. HIV-1 and BLV also encode long non-coding RNAs as antisense transcripts. In this review, we reshape our current understanding of how these antisense transcripts function and how they influence disease pathogenesis.

## Introduction

Viruses that cause persistent infection have strategies to evade host immune responses (1, 2). These viruses that cause chronic infection include human immunodeficiency virus type 1 (HIV-1), hepatitis B virus, hepatitis C virus, Epstein-Barr virus (EBV) and other human herpes viruses, and human T-cell leukemia virus type 1 (HTLV-1). EBV encodes a viral gene homologous to human IL-10, vIL-10, which suppresses the host immune response (3). Nef and Vpu of HIV-1 downmodulate major histocompatibility complex (MHC) class I expression, which leads to impaired cell-mediated immunity against infected cells (4, 5). Another mechanism by which viruses may evade the immune response is to utilize viral functional RNAs, rather than viral proteins, to accomplish some of their purposes, since the host acquired immune system cannot recognize RNAs.

RNA falls into the general classification of messenger RNA (mRNA) and non-coding RNA (ncRNA). NcRNAs include 1) classical ncRNAs such as transfer RNA (tRNA), ribosomal RNA (rRNA), small nuclear RNA (snRNA), and small nucleolar RNA (snoRNA); 2) functional microRNA (miRNA) and 3) long ncRNA (lncRNA) which is generally defined as ncRNA with a length > 200nt (6, 7). The latter two groups have been shown to be biologically functional. These miRNAs and lncRNAs play pivotal roles in diverse biological processes.

In this review, we summarize recent findings on how functional antisense transcripts influence the pathogenicity of retroviruses, focusing on HTLV-1, HIV-1, and bovine leukemia virus (BLV).

## Importance of Viral RNAs in Viral Persistence and Infectivity

Viruses sometimes utilize viral-encoded RNAs, including lncRNA, miRNA, and bifunctional RNA, for replication and persistence *in vivo*. EBV encodes a variety of RNAs that do not encode protein products. Two of these RNAs are EBER1 (167nt) and EBER2 (173nt), which are expressed in all latency types I to III and contain stem-loop RNA hairpins (8, 9). Additionally, two viral non-coding RNA clusters, BamHI-A rightward fragment-derived microRNAs (BART miRNAs) and BamHI-H rightward fragment 1-derived miRNAs (BHRF1 miRNAs), have been identified (9, 10). BART miRNAs are also expressed in all latency types I to III, while BHRF1 miRNA is expressed only in latency type III. BART miRNAs are more strongly expressed in EBV-associated epithelial cells than in B lymphocytes. These viral RNAs regulate the expression of a variety of viral and cellular proteins involved in viral latency, host cell proliferation, and the host immune response. Interestingly, aberrant expression of EBV viral RNAs contributes to oncogenesis in EBV-infected cells (11).

Another DNA virus, human cytomegalovirus (hCMV), contains a virally encoded miRNA, called miR-UL112-1. This miRNA maintains hCMV latency *via* regulation of IE27 (12), and inhibits cytotoxicity by host NK cells (13). Kaposi-sarcoma herpes virus (KSHV) also carries several miRNAs and a lncRNA. The encoded miR-K5, miR-K9 and miR-K10 reactivate KSHV from latent infection by targeting BCLAF-1 (Bcl2-associated factor) (14). The lncRNA encoded by KSHV, polyadenylated nuclear (PAN) RNA, promotes the expression of late viral genes through nuclear RNA transport and interaction with intracellular epigenetic modifiers and viral latent proteins (15).

## HTLV-1 Expresses an Antisense Transcript

**Figure 1:**
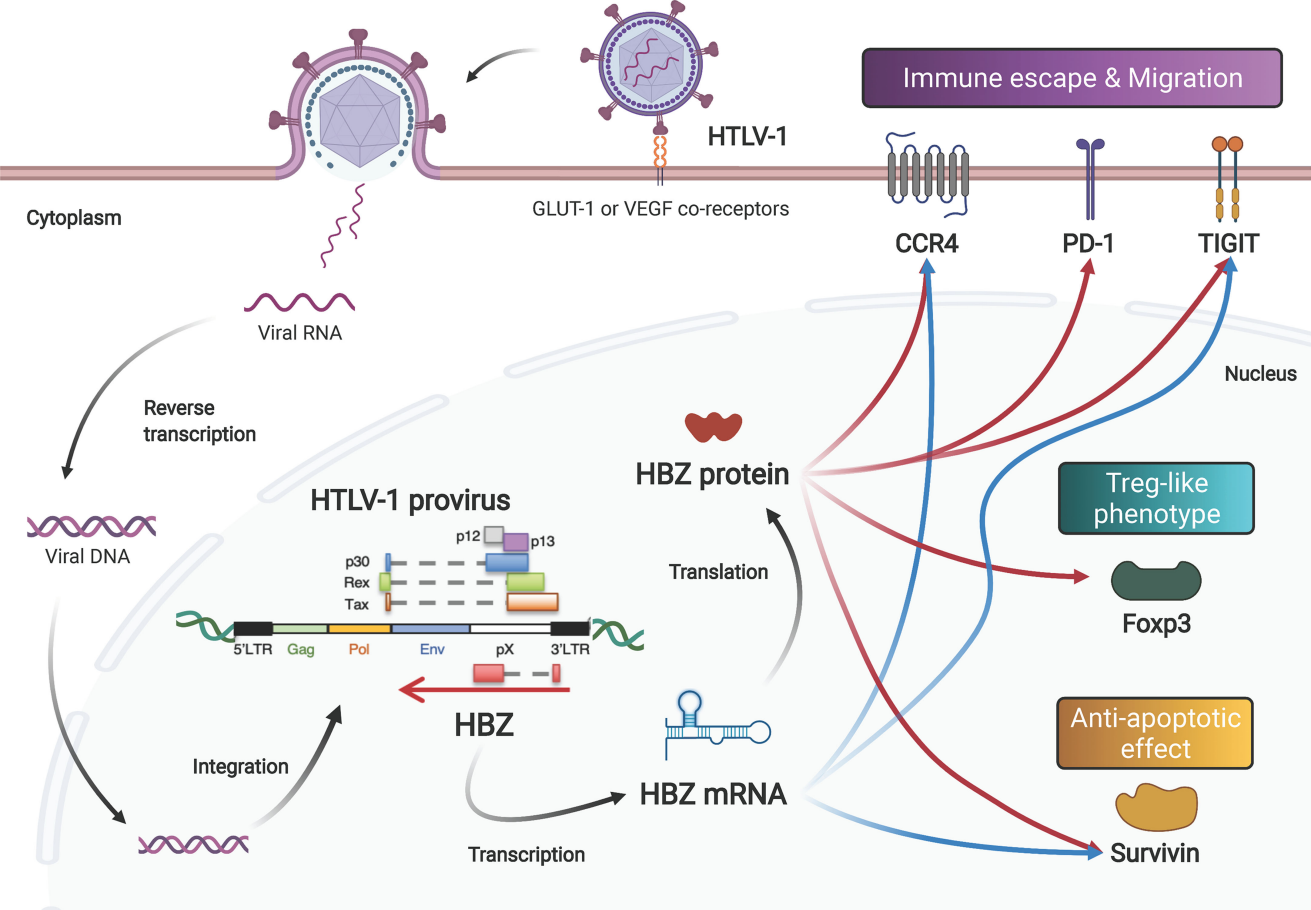
Both the mRNA and protein products of the HTLV-1 *HBZ* gene function to induce proliferation, survival, and phenotype change of infected cells. After infection *via* its receptors (GLUT-1, neuropilin-1 and heparan sulfate proteoglycan), the HTLV-1 genome is integrated into the host’s DNA. HTLV-1 encodes viral genes in the sense and antisense strand of the provirus. The antisense gene, *HBZ*, is transcribed into mRNA, and subsequently translated into protein. Both the mRNA and the protein enhance the expression levels of *CCR4*, *Tigit* and *Survivin*. In addition, HBZ protein enhances transcription of PD-1 and *Foxp3* genes.

HTLV-1 is the first pathogenic human retrovirus to be discovered (16, 17). After the discovery of HTLV-1, HIV-1 was found to be the causative agent of AIDS (18–20). Retroviruses are classified as positive-sense single-stranded RNA viruses. Retroviral genomic RNA is converted into DNA by reverse transcriptase, and the resulting double-stranded DNA is incorporated into the host genome, at which point it is called a provirus (**Figure 1**). In their proviral genomes, retroviruses universally share common viral genes called *gag* (structural protein), *pro* (protease), *pol* (reverse transcriptase) and *env* (envelope protein) which are flanked by the 5’ and 3’ long terminal repeats (LTRs). The LTR has promoter activity in both directions, sense and antisense (21, 22). It has recently been shown that mRNAs and lncRNAs transcribed from the minus strand are also functionally and pathogenically active, and they are the subject of this review.

The two major human retroviruses, HTLV-1 and HIV-1, both target CD4^+^ T cells, but their modes of transmission are completely different (23). For *de novo* infection, HTLV-1 transmits predominantly by cell-to-cell contact (24, 25) and then amplifies a number of retroviral copies in the infected individual by stimulating the proliferation of infected cells (26, 27). In contrast, HIV-1 efficiently infects *via* cell-free viral particles in addition to cell-to-cell contact. Thus, unlike HIV-1, HTLV-1 needs to induce proliferation of the infected cells – yet at the same time, the virus protects the infected cells themselves from being attacked by the host immune system. In this regard, the antisense-encoded gene *HBZ* plays a critical role (28, 29).

## The Virological Significance of *HBZ*, an Antisense Gene of HTLV-1

The *HBZ* gene is encoded in the antisense strand of the provirus. *HBZ* mRNA is transcribed from the 3’ LTR promoter of HTLV-1. *HBZ* was the first retroviral antisense transcript to be identified, in 2002 (30). *HBZ* is also the only retroviral gene that is constantly expressed in all ATL cells (31). *HBZ* promotes the proliferation of T cells, and knockdown of the *HBZ* gene induces cellular death in both HTLV-1-infected and ATL cell lines. Moreover, transgenic mice containing *HBZ* under the control of a CD4^+^ T cell-specific promoter (HBZ-Tg mice) develop systemic inflammation and T-cell lymphoid malignancies (32). These observations show that the *HBZ* gene plays an essential role in the oncogenesis of ATL.

*HBZ* does encode a protein product, and its protein product has many important functions while localizing in the nucleus with speckle-like structures (30, 33) (**Figure 1**). HBZ protein consists of three major domains: an N-terminal transcription activation domain (AD), a central domain (CD), and a C-terminus bZIP domain with a leucine zipper motif (34). The AD includes two LXXLL-like motifs and interacts with the p300/CBP coactivator family (35). Interaction between HBZ and p300 has different effects on different pathways: activation for TGF-β signaling and suppression for the AP-1 pathway. HBZ upregulates Foxp3 expression by activating the TGF-β signaling pathway in a p300-dependent manner; Foxp3 expression gives the HTLV-1-infected cells a Treg-like phenotype (36). While HBZ induces Foxp3 expression, HBZ directly interacts with Foxp3 to impede its DNA-binding activity and function. HBZ also increases the number of induced Treg cells with unstable Foxp3 expression, leading to convert them to Foxp3-negative Treg cells with higher production of IFN-γ (37). Foxp3 is indeed expressed in almost all ATL cases (38). On the other hand, both the CD and bZIP domains harbor nuclear localization signals (NLSs). These NLSs allow HBZ to be retained in the nuclei of infected cells (39). Through its AD and bZIP domains, HBZ binds to a variety of transcription factors of the AP-1 family, which also has a leucine zipper domain (40). Other bZIP transcriptional factors that interact with HBZ bZIP domain are reported as follows: CREB and CREB-2 (41); ATF-1, -2 and -3 (42, 43); C/EBPα and γ (42, 44); MafB (45, 46).

## *HBZ* mRNA Acts as Both Coding and Non-Coding RNA

The antisense *HBZ* gene is transcribed into mRNA and can be translated into HBZ protein. Impressively however, *HBZ* mRNA itself is also functional and pathogenic. We have discovered that an *HBZ* mutant that acts only as mRNA because it cannot be translated into protein (starting-codon ATG converted to TTG) induces T-cell proliferation (31). Furthermore, another *HBZ* mutant with silent mutations for all coding sequence could produce the same protein, but its sequence is different from the wild type, which alters RNA structure. This mutant did not induce proliferation, but rather induced cell death. These findings imply that *HBZ* mRNA itself promotes the proliferation of HTLV-1-infected cells, and additionally, that the expression of HBZ protein without the functional RNA may be toxic to the infected cells. Further analysis based on the predicted stem-loop structures of the native *HBZ* mRNA showed that the first 20nt are important for the growth-promoting activity of *HBZ* mRNA. Moreover, a recent study of *HBZ* mRNA revealed that this antisense transcript can silence sense transcriptions of HTLV-1 *via* displacing TATA box–binding protein (TBP) and RNA polymerase II from the 5’ LTR, thereby contributing to the latency of the virus (47).

In addition to maintaining viral latency and stimulating cell proliferation, *HBZ* mRNA also has an anti-apoptotic effect. *HBZ* mRNA influences transcription of many genes of the cell cycle, proliferation and survival, including the *survivin* gene (48). The *survivin* promoter is activated by *HBZ* mRNA. Survivin belongs to the inhibitor of apoptosis protein (IAP) family that interferes with caspases, the proteolytic components of the apoptotic pathway (49). Interestingly, another group demonstrated that HBZ protein also activates the *suvivin* promoter (50). HBZ protein represses one of the Nuclear Factors Associated with double-stranded RNA (NFAR) proteins, called NF110, which has an antagonistic effect on the *survivin* gene (51). A further study disclosed that the HBZ protein also enhances expression of programmed cell death 1 (PD-1) without impairing T-cell proliferation (52). Taken together, these reports suggest that the mRNA and protein encoded by the *HBZ* gene may complement and support each other’s functions in promoting cell proliferation and preventing apoptosis (**Figure 1**).

*HBZ* is the first viral gene demonstrated to be bifunctional. A recent study reported that a micropeptide translated from lncRNA, *APPLE*, promotes oncogenesis of acute myeloid leukemia by enhancing leukemia-specific translations. This micropeptide is located in ribosomes and functions as an oncoprotein, indicating that APPLE not only functions as lncRNA, but also encodes functional protein (53). Thus, some lncRNAs are bifunctional.

Concerted Gene Regulation by *HBZ* mRNA and Protein Induces a Treg-Like Phenotype and Helps HTLV-1 to Evade Host Immunosurveillance

As discussed, both molecular products of the *HBZ* gene (its mRNA and its protein) activate expression of *survivin*. In addition, these two *HBZ* gene products also target host genes associated with the Treg-like phenotype (46, 54). *CCR4* and *GATA3* are upregulated by both the mRNA and protein products of the *HBZ* gene (**Figure 1**). Both *HBZ* mRNA and its protein are able to induce expression of *GATA3*, and GATA3 in turn bound to GATA3-binding sites within the promoter region of *CCR4*, leading to the activation of the *CCR4* promoter (55). CCR4 is a seven-transmembrane chemokine receptor that is known to be selectively expressed on Treg, T helper 2 (Th2) and cutaneous leukocyte antigen (CLA)-positive memory T cells. Treg cells and skin-homing T cells migrate to their target tissues using CCR4 in a ligand-dependent manner (56). Furthermore, signaling through CCR4 is associated with proliferation of expressing scells along with signaling from CD103 (55). In fact, more than 90% of ATL cases express CCR4 protein on the cellular surface (57). Currently, anti-CCR4 monoclonal antibody (mogamulizumab) therapies are widely practiced in patients with ATL (58, 59) or HAM/TSP (60).

Following this report, the *T-cell immunoreceptor with Ig and ITIM domains (TIGIT)* gene was identified as another gene upregulated by both the mRNA and protein of *HBZ* (**Figure 1**) (61). TIGIT is an immune checkpoint receptor expressed on the surface of Treg cells, cytotoxic T cells and NK cells, as well as tumor-infiltrating T cells (62). TIGIT shows higher affinity to CD155, which is expressed on dendritic cells and tumor cells, than the immune-activating receptor CD226 (also known as DNAM1) on the cytotoxic T cells and NK cells, resulting in suppression of immune activation (63). Signaling through TIGIT suppresses activation through phosphorylation of SHP-2, leading to dephosphorylation of signaling molecules. Expression of TIGIT on tumor-infiltrating T cells results in exhaustion of tumor immunity (64). TIGIT expression on Treg cells enhances their ability to suppress immune responses, especially for the Th1 and Th17 cell subsets, through interaction with CD155 (65). Thus, when *HBZ* mRNA and protein upregulate TIGIT, they can suppress immune responses against HTLV-1. sIndeed, in HBZ-Tg mice, stimulation of CD4^+^ T cells with CD155 enhanced the production of IL-10, an immunoinhibitory cytokine (61). These data suggest that both the mRNA and protein products of *HBZ* alter the immunophenotype of infected cells into a Treg-like phenotype, allowing HTLV-1 to evade host immunosurveillance. Since the *CCR4* and *GATA3* genes are frequently altered, including with gain-of-function mutations, in patients with ATL (66–68), this immune evasion mechanism could also be closely related to the oncogenesis of ATL.

## Constitutively Expressed Antisense Transcripts in Both Leukemic and Nonmalignant BLV-Infected Cells

BLV is closely related to HTLV-1. BLV also belongs to the deltaretrovirus genus and causes leukemia of B lymphocytes. BLV infects B cells of cattle, zebu and water buffalo *in vivo* and causes B-cell persistent lymphocytosis in one-third of infected cattle. Just as a small fraction of people infected with HTLV-1 develop ATL, about 5% of BLV-infected cattle develop fatal B-cell leukemia-lymphoma (69, 70). BLV encodes a transactivator, G4, which is a nonstructural protein resembling Tax of HTLV-1 (71). In addition, BLV encodes miRNAs in the sense strand transcribed by noncanonical RNA polymerase III (72, 73). These miRNAs are constitutively expressed in BLV-induced B-cell leukemia-lymphoma. More intriguingly, deletion of the BLV-derived miRNA cluster reduced viral replication and suppressed leukemia development *in vivo* (74, 75).

Furthermore, recent deep sequencing studies revealed two BLV antisense transcripts, called AS1 and AS2 (76). Of particular interest is AS1, which contains a small open reading frame of 264 bp with ambiguous coding potential; however, the transcript is mainly retained in the nucleus, suggesting that AS1 may play a lncRNA-like role. These antisense transcripts are consistently expressed in both tumor and non-tumor clones, implying an important role for them in the life cycle of BLV and potentially in tumorigenesis. Another high throughput sequencing method revealed that a BLV provirus with a deletion of the 5’ LTR could still induce B-cell persistent lymphocytosis – a phenomenon also reported for HTLV-1 (77). Altogether, recent findings support the virological significance of antisense transcripts transcribed from 3’ LTR of BLV.

## An Antisense Transcript Encoded by HIV-1 and Its Viral Significance

In 1988, a year before the discovery of the *HBZ* gene (21), it was reported that the antisense strand of the HIV-1 genome contained an open reading frame (ORF) that was highly conserved among 12 isolated viral strains in GenBank and encoded a putative protein of 189 amino acid residues, later known as the antisense protein (ASP) (78). The *ASP* gene overlaps the *env* gene of the sense strand. Northern blot analysis detected *ASP* mRNA with a poly-A tail in H9 cells during the acute phase of infection with HIV-1 strain IIIB (79). Moreover, the native promoter of *ASP* was identified within the 3’ LTR, and antibodies to ASP were found in HIV-1 infected patients (80, 81).

In spite of these solid findings, the *ASP* gene had little impact on retrovirologists, since retroviral genes were generally thought to be expressed only from the promoter of the 5’ LTR, and since, especially in the research area of HIV-1, viral genes encoded in the sense strand were under intense investigation. However, the evidence for expression of the antisense transcript was solid, since the promoter, poly-A tail, and protein translation initiation sequence, were conserved in all 12 strains (78). Recently, new discoveries have brought antisense transcription back into the spotlight. Antisense transcriptional activity was reported to be higher in monocyte-derived macrophages and dendritic cells than in activated T cells. These antigen-presenting cells (APCs) with high antisense transcriptional activity did not produce Gag protein (82). A CD8^+^ T-cell-mediated immune response to ASP was revealed by an ASP-specific IFN-γ ELISpot assay, suggesting that antisense transcription and encoded protein are active during the infection and is targeted by host immunosurveillance (83). In a large cohort study of ∼23,000 HIV-1 and simian immunodeficiency virus (SIV) sequences, the *ASP* ORF was present only in Group M viruses, and correlated with the subtype which caused the pandemic (84). These recent findings support the virological significance of the *ASP* gene *in vivo*.

## *ASP* mRNA Regulates HIV-1 Replication Epigenetically

Recently, it has become clear that natural antisense transcripts can repress sense gene expression (85). Indeed, it has been shown that HIV-1 antisense mRNA suppresses gene expression of the sense strand (86). One mechanism of sense-strand repression appears to involve polycomb repressive complex 2 (PRC2), which is mainly composed of EZH2, EED and SUZ12 and modifies chromatin by trimethylating lysine 27 on histone H3 (H3K27me3) to cause transcriptional repression (87). Intriguingly, downregulation of the *ASP* gene has been shown to reduce the recruitment of EZH2 and two other epigenetic-related molecules, DNMT3A and HDAC1, to the HIV-1 5’ LTR (88). A subsequent study clearly showed that *ASP* mRNA associates with PRC2 (89). Ectopic expression of *ASP* mRNA reduced HIV-1 replication and induced viral latency in Jurkat cells. This antisense mRNA was shown to interact with PRC2 and to be recruited to the HIV-1 5’ LTR, increasing the accumulation of the repressive epigenetic mark H3K27me3, while simultaneously decreasing RNA polymerase II and repressing proviral transcription. Taken together, these reports show that the *ASP* gene antisense transcript plays a role in its mRNA form, helping to induce and/or maintain viral latency. To date, little is known about any function of the ASP protein, though the fact that it is highly conserved (including its start codon) suggests that it plays some important role. Thus, we speculate that, like the *HBZ* gene in HTLV-1, the *ASP* gene in HIV-1 may encode a bifunctional mRNA.

## Insufficient Polyadenylation Confers Nuclear Retention on These Human Retroviral Antisense mRNAs

Presumably, only the small amount of HBZ protein found in the cell would be subject to host immunosurveillance. Furthermore, the immunogenicity of HBZ protein is very low (90). The mRNA can not be recognized by CTL. Thus, *HBZ* carries out many of its functions in “stealth” forms and locations. We have recently discovered that the antisense mRNAs of both HTLV-1 and HIV-1 are normally localized primarily in the nucleus (91). Nuclear retention of *HBZ* mRNAs in primary cells from ATL and HAM patients was reported (92). However, *HBZ* mRNA was present in the cytoplasm of cells in which *HBZ* mRNA was overexpressed, suggesting that polyadenylation and promoter activity were involved in its localization. Compared to *HBZ* overexpressing cells, a length of poly-A tail was reduced and 3’ LTR promoter activity was weaker in HTLV-1 infected cells. These findings were also confirmed for *ASP* in HIV-1. Furthermore, there is no degradation of *HBZ* mRNA when HTLV-1-infected cells are treated with cordycepin, an inhibitor of polyadenylation although deadenylation is associated with mRNA decay (93). Thus, due to the low transcriptional activity of the 3’ LTR, the antisense mRNAs are often insufficiently polyadenylated, in other words, shorter lengths poly-A tail compared to sense mRNAs, resulting in their tendency to be retained in the nucleus where they can affect the transcription of host genes. Polyadenylation is a critical step for stabilization and transition of mRNA from nucleus to cytoplasm (94). Therefore, *HBZ* mRNA is localized in the nucleus due to insufficient polyadenylation, which is a mechanism commonly observed in nuclear-localized lncRNAs (95).

**Figure 2:**
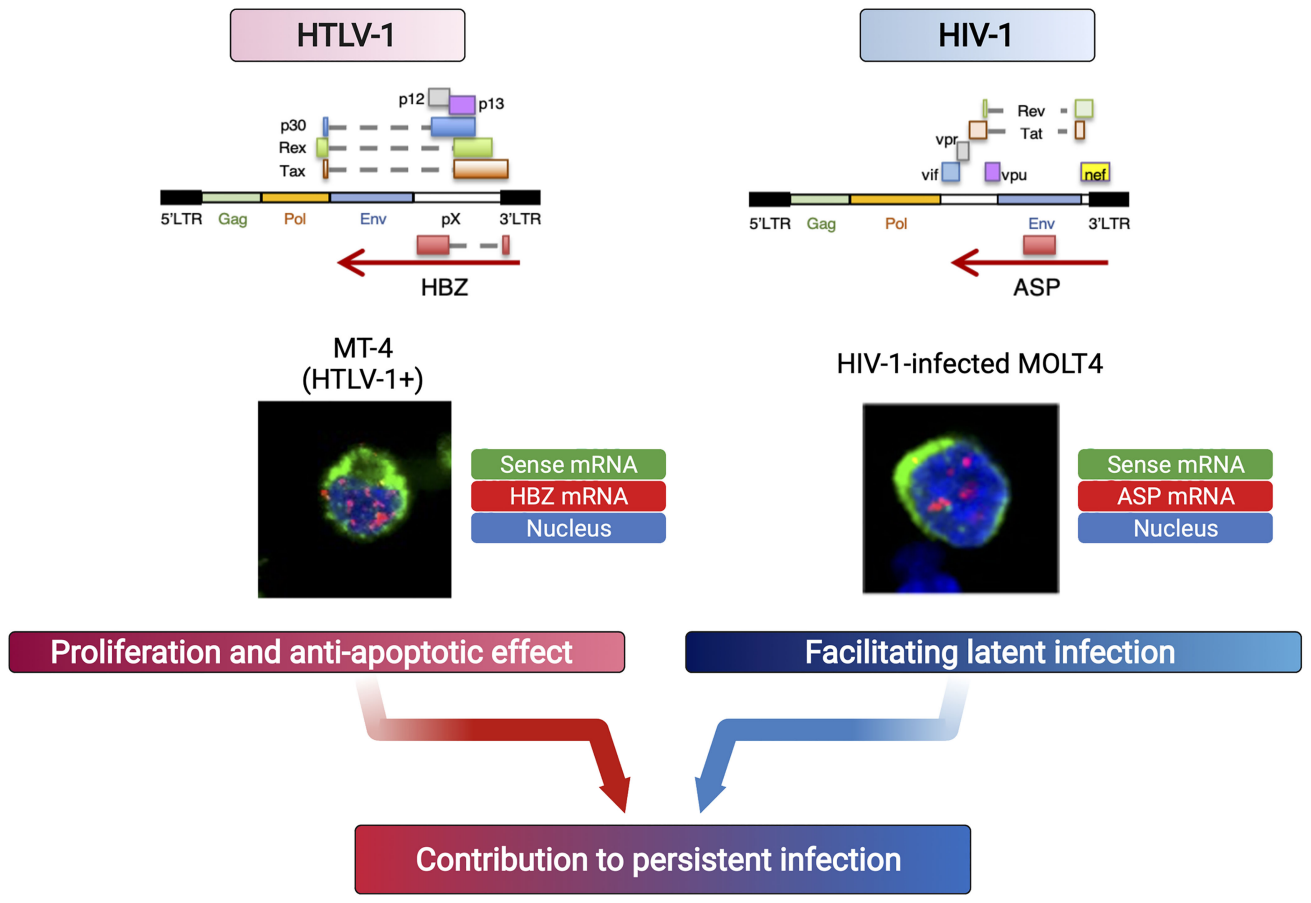
Retroviral antisense mRNAs persist in the nucleus and function like lncRNAs. Due to insufficient polyadenylation, human retroviral antisense transcripts remain in the nucleus. Each mRNA acts differently to affect the survival of the infected cells (HTLV-1) or viral latency (HIV-1, HTLV-1), leading to persistent infection (91).

Both *HBZ*, the antisense gene of HTLV-1, and *ASP*, the antisense gene of HIV, encode mRNAs that are retained in the nucleus and contribute to the persistence of infection by functioning in the proliferation of infected cells (for HTLV-1) and in the latency of the virus (for HIV-1) (**Figure 2**). These antisense mRNAs encode protein and yet function as RNA with more than 200nt, suggesting that they are lncRNA-like RNA molecule or “coding non-coding RNA (cncRNA)”. These retroviral antisense transcripts exert their function in the nucleus by regulating gene transcription, including through epigenetic mechanisms, but the “end goals” of this gene regulation differ, based on what is appropriate to each retrovirus in establishing and maintaining infection.

## Concluding Remarks

Retroviruses cleverly evade host immunosurveillance and expand their own numbers by intricate mechanisms that include the persistent expression of viral antisense transcripts. These transcripts are disproportionally retained in the nucleus and have lncRNA-like functions. These functions contribute to the persistence of the virus, and to its pathological effects. Moreover, the antisense transcripts of HTLV-1, and possibly HIV-1, can function in both molecular forms, mRNA and protein, suggesting that they are bifunctional.

## Author Contributions

KT and MM wrote this review article. All authors contributed to the article and approved the submitted version.

## Funding

This research is supported by a grant from the Project for Cancer Research And Therapeutic Evolution (P-CREATE) (20cm0106306h0005 to MM), the Research Program on Emerging and Re-emerging Infectious Diseases (20fk0108088h0002 to MM) from the Japan Agency for Medical Research and Development (AMED), and JSPS KAKENHI (19H03689 to MM). This study was also supported in part by the JSPS Core-to-Core Program A, Advanced Research Networks.

## Conflict of Interest

The authors declare that the research was conducted in the absence of any commercial or financial relationships that could be construed as a potential conflict of interest.

## Publisher’s Note

All claims expressed in this article are solely those of the authors and do not necessarily represent those of their affiliated organizations, or those of the publisher, the editors and the reviewers. Any product that may be evaluated in this article, or claim that may be made by its manufacturer, is not guaranteed or endorsed by the publisher.
